# Risk factors for house-entry by culicine mosquitoes in a rural town and satellite villages in The Gambia

**DOI:** 10.1186/1756-3305-1-41

**Published:** 2008-10-21

**Authors:** Matthew J Kirby, Philippa West, Clare Green, Momodou Jasseh, Steve W Lindsay

**Affiliations:** 1 School of Biological and Biomedical Sciences, Durham University, Science Laboratories, South Road, Durham, DH1 3LE, UK; 2 Essex Health Protection Unit, Collingwood Road, Witham, Essex, CM8 2TT, UK; 3 Centre for Infectious Diseases & International Health, Windeyer Institute of Medical Research, UCL, 46 Cleveland Street, London, W1T 4JF, UK; 4 Medical Research Council Laboratories, Fajara, P.O. Box 273, The Gambia

## Abstract

**Background:**

Screening doors, windows and eaves of houses should reduce house entry by eusynanthropic insects, including the common African house mosquito *Culex pipiens quinquefasciatus *and other culicines. In the pre-intervention year of a randomized controlled trial investigating the protective effects of house screening against mosquito house entry, a multi-factorial risk factor analysis study was used to identify factors influencing house entry by culicines of nuisance biting and medical importance. These factors were house location, architecture, human occupancy and their mosquito control activities, and the number and type of domestic animals within the compound.

**Results:**

40,407 culicines were caught; the dominant species were *Culex thalassius*, *Cx. pipiens s.l.*, *Mansonia africanus*, *M. uniformis *and *Aedes aegypti*. There were four times more *Cx. pipiens s.l. *in Farafenni town (geometric mean/trap/night = 8.1, 95% confidence intervals, CIs = 7.2–9.1) than in surrounding villages (2.1, 1.9–2.3), but over five times more other culicines in the villages (25.1, 22.1–28.7) than in town (4.6, 4.2–5.2). The presence of *Cx. pipiens s.l. *was reduced in both settings if the house had closed eaves (odds ratios, OR town = 0.62, 95% CIs = 0.49–0.77; OR village = 0.49, 0.33–0.73), but increased per additional person in the trapping room (OR town = 1.16, 1.09–1.24; OR village = 1.10, 1.02–1.18). In the town only, *Cx. pipiens s.l. *numbers were reduced if houses had a thatched roof (OR = 0.70, 0.51–0.96), for each additional cow tethered near the house (OR = 0.73, 0.65–0.82) and with increasing distance from a pit latrine (OR = 0.97, 0.95–0.99). In the villages a reduction in *Cx. pipiens s.l. *numbers correlated with increased horses in the compound (OR = 0.90, 0.82–0.99). The presence of all other culicines was reduced in houses with closed eaves (both locations), with horses tethered outside (village only) and with increasing room height (town only), but increased with additional people in the trapping room and where cows were tethered outside (both locations).

**Conclusion:**

The findings of this study advocate eave closure and pit latrine treatment in all locations, and zooprophylaxis using horses in rural areas, as simple control measures that could reduce the number of culicines found indoors.

## Background

Disease control strategies that aim to reduce human-vector contact must consider the risk factors that influence where and when this contact occurs. The house is a common site of human-vector contact and this is reflected in the high degree of endophily and endophagy exhibited by important vector species, such as the malaria-transmitting mosquitoes *Anopheles gambiae sensu stricto *Giles and *An. funestus *Giles over much of Africa[1], *Culex pipiensquinquefasciatus *Say, a domestic biting nuisance worldwide and a vector of lymphatic filariasis in tropical Africa, Southeast Asia, Western Pacific and the Americas[2,3], and *Aedes aegypti *L. in West Africa, where it is a vector of yellow fever, dengue and Chikungunya viruses[4]. House-entering behaviour has evolved in some mosquito species and, more specifically, within some species populations but not others [4,5]. This behaviour may partly be a consequence of their anthropophily; these species have evolved with their human hosts and in human-made environments[6]. For example, 'domestic' *Ae. aegypti *breed year round in clay jars maintained indoors for water storage [7]. The attractiveness and ease of entry into a house by mosquitoes is affected by both structural factors and social practises. A recent study in a semi-rural area of The Gambia showed that *An. gambiae sensu lato *are more likely to enter houses with open eaves, mud brick walls and many occupants, whilst burning churai, a local incense, reduced house entry [8].

Studies that use entomological techniques such as light traps or knockdown catch, to monitor anopheline populations as part of malaria surveillance and risk assessment, will usually also trap a large number of culicine mosquitoes. Often the data relating to the culicine portion of the catch is not analysed or not published, yet some of the culicines trapped serve as disease vectors and major nuisance biters. Here we have assessed putative risk factors that determine the level of human exposure indoors to potential vectors of pathogens other than malaria. A multi-factorial risk factor analysis study was designed to highlight important spatial, compound-, house- and mosquito control-related parameters that affect house entry of culicine vectors and species that are a biting nuisance in The Gambia.

## Methods

### Study area

The study area was situated approximately 170 km from the mouth of the River Gambia and covered 70 km^2 ^of the North Bank Division in The Gambia, an area of open Sudan savanna vegetation. The climate consists of a single rainy season from June to October followed by a long dry season. Details of the study site and population are given by Kirby *et al.*[8]. Briefly, the study area comprised 976 houses; 539 houses in 11 residential blocks in Farafenni town (UTM coordinates: 1500200N, 435500E) and 437 in 16 villages (designated as individual blocks) located to the south and east, within 5 km of the town. A house was defined as a discrete building of one or more rooms and at least one bedroom, occupied by at least one person. Houses in both the town and villages were usually arranged in familial compounds demarcated by a low fence or wall. Approximately 70% of all compounds contained a pit latrine. The study population comprised 5,848 people dominated by three ethnic groups; Mandinka (38%), Wollof (31%) and Fula (23%).

### Mosquito collections

976 houses were each sampled on a single night between 17 August and 25 October 2005. A CDC miniature light trap (Model 512, John W. Hock Co., Gainesville, FL) was positioned 1 m above the ground and within 1–2 m of the foot end of a bed protected with a new untreated bednet provided on that night only. If the trapping room contained multiple beds, then the other room occupants were encouraged to use their existing bednets. If they had none, additional new bednets were provided for that night. Light traps operated from 19:00 to 07:00 the following morning. Light traps were also hung in four sentinel houses, two in Farafenni, one in a village to the east, Kunjo, and one in a village to the south, Duta Bulu, on every night of collection in order to adjust for night-to-night variation in mosquito catches. These houses were each occupied by a single adult male that slept under an untreated bednet for the duration of the rainy season.

Mosquitoes were killed by freezing at -25°C for two hours and identified using morphological criteria [9]. Approximately 0.5% of the *Cx. pipiens *complex caught in the traps were chosen for species identification by PCR analysis[10].

### Putative risk factors

The spatial parameters recorded were house location (block and urban/rural). The compound parameters documented were the number of cows and horses in the compound between 19.00 and 07.00, and the distance (m) to the nearest pit latrine from the external wall of the trapping room. Variables of house structure recorded were house external length and width, trapping room internal length, width and height, size of eave gap (all cm), eaves open/closed, roofing material, wall material, number of storeys, presence or absence of ceilings, screening, or electric lights. The variables of occupants recorded were age, sex and ethnicity of those sleeping in the bed nearest the light trap, the number of children sleeping in the trapping room and all other rooms on the night of trapping, the number of adults sleeping in the trapping room and all other rooms on the night of trapping. The trapping room volume, house volume, number of people/house volume and number of adult equivalents/house were derived from the house structure and occupant variables recorded. Variables of mosquito control noted were use of mosquito coils, local incense (churai), insecticide spray and insecticide-treated nets within the trapping room and all other rooms on the night of trapping.

### Statistical analysis

Mosquito catches from individual houses were analysed as unadjusted counts, and also as adjusted counts to account for the large night to night variation observed in the mosquitoes in the sentinel houses. The study house mosquito catch numbers were divided by the sentinel house geometric mean mosquito catch for that night.

For some variables there were too few values to determine whether they affected mosquito house entry or not (evidence of screening n = 23; electric lights, n = 7; multi-storey housing, n = 1) and were not analysed further.

The unconditional variance of the total culicine mosquito counts (standard deviation = 72.4) in the 976 houses was much larger than the mean (mean = 41.7), therefore a negative binomial model was used that, unlike the Poisson model, accounts for the excess variation.

The association of each risk factor with mosquito catch size was individually assessed with univariate analyses. If the univariate regression model for a given covariate resulted in a p-value greater than 0.1 then this covariate was excluded from the multivariate investigations. Where plausible, the risk factors were then categorized into five groups within which there was a high degree of correlation. House location, and distance to nearest pit latrine from external wall of trapping room, were incorporated in the model, but not as part of a specific group. The five groupings were:

1. Number of children in the trapping room, people in the trapping room, children in the house, people in the house;

2. House external length and width and volume, trapping room internal length, width, height and volume, number of people/house volume, number of adult equivalents/house;

3. Wall type, roof type, eaves (presence/absence), size of eave gap;

4. Use of mosquito coils, local incense (churai), insecticide spray and insecticide-treated nets;

5. Number of cows and horses within the compound;

Generalized linear model multivariate analyses (GLM) were conducted treating *Cx. pipiens s.l. *count data and all other culicine numbers as two separate dependent variables, both fitted to a negative binomial distribution with a log link function. *Cx. pipiens s.l. *merited separate analysis as they were by far the most common disease vector of all the culicines caught. The analyses were conducted in two steps: firstly the association of the dependent variables with groups of risk factors described above was investigated. This eliminated some of the risk factors and represented a group of highly correlated variables by possibly just one variable. In step II all significantly associated variables with the outcome from step I as well as those that did not fit in a group were included in the model. Variables were retained in this model if their association with mosquito count was significant (p < 0.05). Urban/rural classification (location) is a known important and overriding factor and, therefore, the data set was analyzed as a whole but also split into two sets by location. Thus the final multivariate model was built six times for each dependent variable; with location in the model, for urban houses only and for rural houses only, in each case with and without adjustment for the sentinel catch.

A Mann-Whitney U test was used to look for an urban-rural difference in catch size and also wall height.

All analyses were conducted using SPSS version 15.0 (SPSS Inc., Chicago, IL) and Epi Info™ version 3.4.3 (Center for Disease Control, Atlanta, GA)

### Ethics

Ethical approval for this study was given by the Gambian Government and Medical Research Council Laboratories Joint Ethical Committee and the Ethics Advisory Committee of Durham University. Verbal and written consent was given by the participants prior to the start of the study.

## Results

### Mosquito numbers

106,536 mosquitoes were caught in 976 light trap collections during the 2005 rainy season, of which 40,407 (38%) were culicines and 66,129 (62%) were anophelines. Only three traps failed during the period; those houses were re-sampled within the sampling timeframe. The dominant species were *Cx. thalassius *(45%), *Cx. pipiens s.l. *(29%), *Mansonia africanus *and *M. uniformis *(18% combined) and *Ae. aegypti *(2%). Other species recorded included *Ae. vittatus*, *Ae. fowleri*, *Cx. tigripes *and *Coquillettidia metallica*. The prior assumption that the majority of *Cx. pipiens s.l.*were *Cx. pipiens quinquefasciatus *was corroborated by PCR. Of 61 specimens tested, 58 (95%) were *Cx. pipiens quinquefasciatus *and 3 (5%) were *Cx. pipiens pipiens*.

### Vector risk factors

Step I of the GLM revealed 8 variables that were significantly associated with the outcome measures: these were location (town/village), roof type, eaves (open/closed), distance to nearest pit latrine, room height, number of people in the room, number of horses tethered in the compound at night and the number of cows tethered in the compound at night. The multivariate analysis on the data unadjusted or adjusted for sentinel house mosquito counts gave fairly similar results (see additional file 1: table 1). Thus the results derived from the unadjusted values are presented.

There were four times as many *Cx. pipiens s.l. *in Farafenni town than in the villages (geometric mean/trap/night in town = 8.1, 95% confidence intervals, CIs = 7.2–9.1; villages = 2.1, 1.9–2.3, Z = -13.9, p < 0.001). By contrast, the numbers of all other culicines caught were over five times lower in town than in the villages (town = 4.6, 4.2–5.2; villages = 25.1, 22.1–28.7, Z = -17.4, p < 0.001). *Cx. pipiens s.l. *represented 69% of all culicines caught in the town but only 6% in the villages.

There were several risk factors common to both the town and the villages (see additional file 1: table 1). The numbers of *Cx. pipiens s.l. *and the total of all other culicines increased per additional person in the trapping room (odds ratios, OR, and 95% confidence intervals for town = 1.16, 1.09–1.24 and 1.22, 1.13–1.30 respectively; OR village = 1.10, 1.02–1.18 and 1.18, 1.11–1.26) but were reduced if the house had closed eaves (OR town = 0.62, 0.49–0.77 and 0.76, 0.61–0.96 respectively; OR village = 0.49, 0.33–0.73 and 0.51, 0.36–0.72).

**Figure 1:**
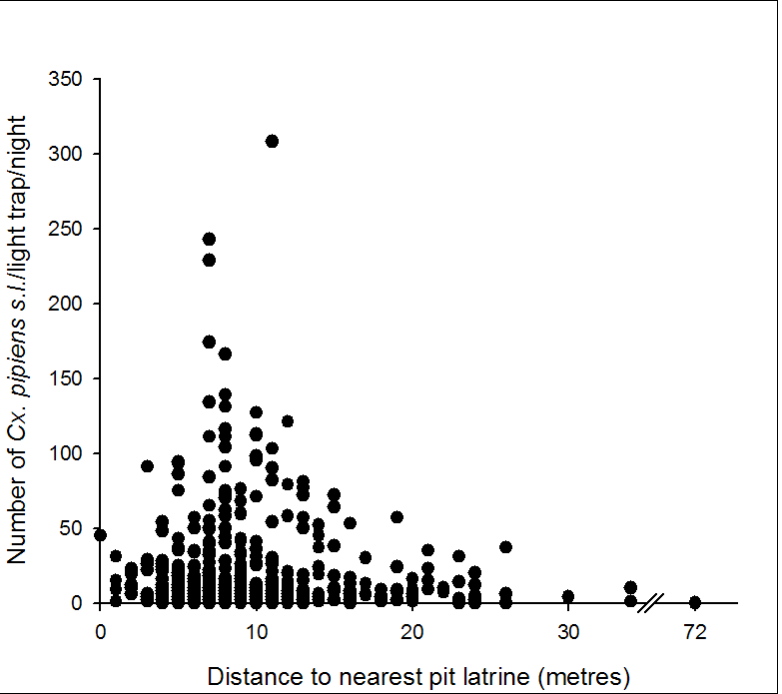
Relationship between the number of *Cx. quinquefasciatus *caught from houses in Farafenni and the distance to the nearest pit latrine.

In the town only, *Cx. pipiens s.l. *numbers decreased with increasing distance from a pit latrine (OR = 0.97, 0.95–0.99, fig 1) and in houses with a thatched roof (OR = 0.70, 0.51–0.96). The presence of all other culicines was reduced with increasing room height (OR = 0.995, 0.992–0.999). The average wall height in Farafenni houses was significantly higher (median and inter-quartile range = 235, 216–257 cm) than in village houses (203, 190–221 cm, Z = -14.6, p < 0.001).

A complex relationship was uncovered between the presence of domestic animals tethered in the compound at night and the number of mosquitoes trapped indoors (see additional file 1: table 1). The presence of cows was associated with a decreased risk of exposure to *Cx. pipiens s.l. *in town only (OR = 0.73, 0.65–0.82) but with an increased risk of exposure to all other culicines in both town and village settings (OR town = 1.24, 1.11–1.38; OR village = 1.32, 1.15–1.53). In the villages the presence of horses was associated with a decreased risk of exposure to both *Cx. pipiens s.l. *and all other culicines (OR = 0.90, 0.82–0.99 and 0.90, 0.84–0.98 respectively) yet no relationships between horses and mosquito numbers in the town were observed. This may be due to the low number of horses present in the town (only 5.6% of compounds have any horses) compared to the villages (49.8%). In the villages there were over three times more horses than cows, but there were twice as many cows to horses in the town.

The use of churai, mosquito sprays, coils and the presence of an insecticide-treated bed net in a bedroom did not reduce the number of mosquitoes caught, though prevalence of use of each of these vector control activities was low (25%, 1%, 13% and 18%).

## Discussion

In this study culicines represented a substantial fraction of the average light trap contents: though there were more anophelines than culicines in all months of the trapping period, the culicine proportion was never smaller than 25% of the total monthly catch. The density of culicines is important as a contributor to the overall biting nuisance and its impact on malaria control. For example, it has been shown that a biting nuisance threshold of 10–15 mosquitoes/person/night exists, irrespective of mosquito species, below which bed nets are not generally used in Gambian villages [11]. Culicines are also important as transmitters of disease. The dominant culicine species were *Cx. thalassius*, *Cx. pipiens s.l.*, *M. africanus*, *M. uniformis *and *Ae. aegypti*. With the exception of *Cx. thalassius*, all of these species are potential vectors in this setting. *Cx. pipiens quinquefasciatus *is probably the most common man-biting culicine in Africa[12,13] and has been shown to be a vector of *Wuchereria bancrofti *[14] and Chikungunya [15] across much of the continent. *Ae. aegypti *is a vector of dengue, urban yellow fever and chikungunya in Cote d'Ivoire[4] whilst *M. africana *and *M. uniformis *have been shown to be vectors of lymphatic filariasis, Rift Valley Fever and West Nile Virus in nearby Senegal[16].

The results presented here identify some of the many factors that govern the risk of exposure to culicine vectors, particularly at the compound and household level. The urban-rural difference in catch numbers and proportion of *Cx. pipiens s.l. *may be explained by the availability of breeding sites, if one accepts that *Cx. pipiens quinquefasciatus *is the dominant member of the complex here. *Cx. pipiens quinquefasciatus *prefer breeding in household sewage systems, soakage pits, septic tanks and storm drains[17,18], structures more common to urban areas. This is also reflected in the relationship between *Cx. pipiens quinquefasciatus *and the distance to the nearest pit latrine in Farafenni. *Cx. pipiens quinquefasciatus *has a very short flight range[19] and is likely to be caught not far from its breeding sites. 'Domestic' *Ae. aegypti *can breed year round in clay jars maintained indoors for water storage[7] which may account for the greater prevalence of this species in town (22% v 18%). By contrast *Cx. thalassius *breeds in brackish water and is common to pools in the mangrove swamps in The Gambia[20], while *M. africana *and *M. uniformis *breed in natural swamp areas and rice fields with emergent vegetation such as water lilies and water lettuce[21,22]. The majority of these types of breeding site are found on the flooded alluvial plains bordering the River Gambia[23,24]. Though the distance of each study house to the nearest larval habitat was not measured, it is the case that the rural settlements are closer to the alluvial plainsand therefore are exposed to more mosquitoes than those situated on the edge and in the middle of Farafenni town, further away from these sites[8,25].

At the compound level, in the villages only, it was found that the presence of horses tethered near the house at night was associated with lower numbers of mosquitoes in the trapping room, but that cows increased the likelihood of culicinesother than *Cx. pipiens s.l.*, entering the house. The presence of smaller domestic animals was not recorded as these were usually kept further from the houses or outside the compound at night. The presence of livestock has previously been shown to attract culicines into a compound[26]. These findings are of significance to mosquito control since zooprophylaxis, the use of large domesticated animals to reduce biting frequencies, has been successful against culicines in some parts of the world[27,28]. Nevertheless, caution should be exercised in recommending zooprophylaxis against culicines, particularly in areas where they are vectors of zoonotic arboviruses that require amplification in domestic animalsOur findings suggest horses may be an important bloodmeal for many culicines here but that cattle, probably through production of CO_2_, act as attractants but not bloodmeals for the culicines found, with the exception of *Cx. pipiens s.l.*. However, blood meal analysis or host choice studies are needed to confirm this. Horses have been shown to reduce culicine house entry in another study in this area[29]. The reasons for the attractiveness of horses may be partly due to the high bed net usage in The Gambia[30] making it difficult for a blood-questing mosquito to locate a human host, as well as the unusually high density of equines in the Senegambia region[31].

Both cattle and humans are primary blood meal sources for *Cx. pipiens quinquefasciatus *in western Kenya[12], which feed opportunistically from humans when they enter houses but largely from cattle outside [32]. It seems plausible that the *Cx. pipiens quinquefasciatus *in this study behaved similarly. That is, they are more catholic in their feeding behavior than reported from other parts of the world e.g. in south India[33].

Within the house the important variables were the status of the eaves, the height of the walls, the roofing material and the number of occupants. Open eaves are the main route by which many mosquitoes, including *Mansonia spp*, enter homes, and closing eaves has been shown to reduce the number of mosquitoes entering a house[34,35]. *Mansonia spp *fly at a very low (<0.15 m) height above the ground[36] and so it seems that they must fly upwards when encountering a vertical wall surface, following the cues emanating from the eaves and becoming channeled indoors through the open eaves by the overhanging roof. This behavior is a common trait of endophagic mosquitoes and probably hasa genetic basis. For example, house entering by *Ae. aegypti *in Kenya was shown to be a characteristic of an indoor form[37] and genetically determined[5]. The importance of eaves to *Cx. pipiens quinquefasciatus *house entry is less clear. For example indoor-resting density was suppressed by 61–96% by deltamethrin-impregnated curtains hung in the eaves and doorways of huts in Southern India[38], but in Trinidad door and window curtains and eave treatment were not related to the number of *Cx. pipiens quinquefasciatus *caught indoors[39].

Wall height had a weak affect on mosquito numbers caught indoors and was only associated significantly with the other culicines group caught in town. *Cx. thalassius *was the dominant mosquito in that group and has been shown to be prevented from entering houses by increasing wall height. For example, a 62% decrease in *Cx. thalassius *house entry was observed between a hut with a 60 cm wall compared to one with a 172 cm wall[35]. The average wall height in Farafenni houses was significantly higher and the minimum-maximum range greater than in village houses which may partly explain why the trend was not observed in the villages.

Thatched roofs are typically associated with open eaves and poorer quality houses in this setting so it is surprising that they are linked with a reduction in *Cx. pipiens s.l.*, albeit in town houses only. Metal roofed houses are more permanent structures, and it has been reported elsewhere that *Culex spp *are more abundant in permanent houses (typically houses with cement walls and metal roofs) compared to traditional thatched homes [39-41]. Why this should be the case is unclear.

Routes of entry into houses are recognized by olfactory cues i.e. the carbon dioxide and body odors emanating from the hosts inside[42]. That more culicines are found in rooms with higher numbers of people is unsurprising since it has been shown that odours emanating from individuals attract mosquitoes towards a human host[40,43]. An increased number of occupants are likely to increase the attractiveness of a house to culicines. Occupant number will increase at night as people go to bed, and this coincides with periods of high activity in two of the most common species caught in this study. *Cx. pipiens quinquefasciatus *is active throughout the night in many locations [3], with peak activity from 22–23:00 h[44], and *M. uniformis *tends to feed indoors post-dusk and leave the subsequent night for outdoor resting sites[45]. Furthermore, while *Ae. aegypti *is typically considered a diurnal species, in Côte d'Ivoire it has shown persistent indoor biting activity during the night up to 06:00 h with peak activity at 00:00 h both inside and outside [4]. The age, sex and ethnicity of the occupants had no obvious effects in this study. Pregnancy has been identified as a risk factor for attractiveness to *Mansonia spp*[46] but was not recorded here.

## Conclusion

Because there may be unmeasured biological or social confounders operating at a scale not explored here, caution is needed before interpreting these results as indicating causal associations, and therefore it is important that these associations be tested by rigorous experimental trials in which each variable can be isolated. Equally it is important to note that much of the culicine-borne disease transmission and biting nuisance will occur away from the home owing to the opportunistic feeding behavior that is characteristic of some of the species captured here. As a result, culicine control campaigns should not ignore biological or chemical approaches to reduce breeding sites. Nevertheless, it is likely that the household factors shown here to be statistically associated with risk of culicine house-entry are relevant to disease transmission. Therefore, these results suggest that closing eaves, covering pit latrines or treating them with polystyrene beads, and zooprophylaxis using horses in rural areas, are simple household control measures that could reduce the number of culicines found indoors.

## Competing interests

The authors declare that they have no competing interests.

## Authors' contributions

MK coordinated the fieldwork, data collection and sample identification, and drafted the manuscript. PW assisted the data collection and sample identification. CG carried out the PCR. MJ participated in the design of the study and provided access to the demographic database. SWL conceived of the study, participated in its design and coordination and helped to draft the manuscript. All authors read and approved the final manuscript.

## Supplementary Material

Additional file 1 Table 1. Association between mosquito counts and potential risk factors as measured in odds ratios (OR) from negative binomial general linear multivariate models. Click here for file
