# Supplementary material for: Risk factors for house-entry by culicine mosquitoes in a rural town and satellite villages in The Gambia
Source: Parasit Vectors. 2008 Oct 21;1:41. doi: 10.1186/1756-3305-1-41 (PMC2584634; doi:10.1186/1756-3305-1-41)
Supplement: Additional file 1 — Table 1. Association between mosquito counts and potential risk factors as measured in odds ratios (OR) from negative binomial general linear multivariate models. [file 1756-3305-1-41-S1.pdf]

Table 1. Association between mosquito counts and potential risk factors as measured in odds ratios (OR) from negative binomial general linear multivariate models. Significant values (<0.05) are highlighted in bold.

	Unadjusted *Cx. pipiens s.l.* (n=515)		Adjusted *Cx. pipiens s.l.* (n=515)		Unadjusted all other culicines (n=514)		Adjusted all other culicines (n=514)	
Town houses								
	OR (95%CI)	p	OR (95%CI)	p	OR (95%CI)	p	OR (95%CI)	p
Horses	1.19 (0.96-1.47)	0.12	0.99 (0.80-1.22)	0.93	1.21 (0.93-1.59)	0.16	0.96 (0.75-1.25)	0.78
Cows	0.73 (0.65-0.82)	**<0.001**	0.88 (0.79-0.97)	**0.01**	1.24 (1.11-1.38)	**<0.001**	1.43 (1.29-1.60)	**<0.001**
Eaves								
open	1		1		1		1	
closed	0.62 (0.49-0.77)	**<0.001**	0.70 (0.56-0.87)	**0.001**	0.76 (0.61-0.96)	**0.03**	0.84 (0.67-1.03)	0.10
Roof								
metal	1		1		1		1	
thatch	0.70 (0.51-0.96)	**0.03**	0.72 (0.53-0.96)	**0.03**	1.19 (0.88-1.62)	0.26	1.27 (0.96-1.69)	0.10
Distance to latrine	0.97 (0.95-0.99)	**0.011**	0.98 (0.95-0.99)	**0.007**	0.99 (0.98-1.01)	0.24	0.99 (0.98-1.01)	0.50
People in room	1.16 (1.09-1.24)	**<0.001**	1.16 (1.06-1.28)	**0.002**	1.22 (1.13-1.30)	**<0.001**	1.17 (1.10-1.25)	**<0.001**
Room height	1.00 (0.99-1.01)	0.17	1.004 (1.001-1.008)	**0.018**	0.995 (0.992-0.999)	**0.017**	0.99 (0.98-1.00)	0.58
Village houses								
Horses	0.90 (0.82-0.99)	**0.027**	0.86 (0.79-0.94)	**0.001**	0.90 (0.84-0.98)	**0.01**	0.87 (0.81-0.95)	**0.001**
Cows	1.14 (0.99-1.31)	0.07	1.34 (1.19-1.52)	**<0.001**	1.32 (1.15-1.53)	**<0.001**	1.31 (1.14-1.52)	**<0.001**
Eaves								
open	1		1		1		1	
closed	0.49 (0.33-0.73)	**<0.001**	0.60 (0.42-0.86)	**0.006**	0.51 (0.36-0.72)	**<0.001**	0.56 (0.40-0.80)	**0.001**
Roof								
metal	1		1		1		1	
thatch	0.94 (0.66-1.33)	0.71	1.04 (0.75-1.43)	0.82	0.74 (0.54-1.00)	0.051	0.68 (0.50-0.92)	**0.013**
Distance to latrine	1.00 (0.99-1.00)	0.08	0.994 (0.990-0.998)	**0.007**	1.000 (0.996-1.004)	0.86	1.00 (0.996-1.004)	0.91
People in room	1.10 (1.02-1.18)	**0.012**	1.08 (1.01-1.16)	**0.02**	1.18 (1.11-1.26)	**<0.001**	1.14 (1.07-1.21)	**<0.001**
Room height	1.00 (0.99-1.00)	0.46	0.994 (0.988-0.999)	**0.03**	1.000 (0.995-1.006)	0.97	0.997 (0.992-1.003)	0.33
